# Tripartite assembly of RND multidrug efflux pumps

**DOI:** 10.1038/ncomms10731

**Published:** 2016-02-12

**Authors:** Laetitia Daury, François Orange, Jean-Christophe Taveau, Alice Verchère, Laura Monlezun, Céline Gounou, Ravi K. R. Marreddy, Martin Picard, Isabelle Broutin, Klaas M. Pos, Olivier Lambert

**Affiliations:** 1 Université de Bordeaux, CBMN UMR 5248, Bordeaux INP, IECB, Pessac F-33600, France; 2 CNRS, CBMN UMR 5248, Pessac F-33600, France; 3 Laboratoire de Cristallographie et RMN Biologiques, UMR 8015, CNRS, Université Paris Descartes, Faculté de Pharmacie, 4 Avenue de l'Observatoire, Paris 75006, France; 4 Institute of Biochemistry, Goethe-University Frankfurt, Max-von-Laue-Str. 9, D-60438 Frankfurt am Main, Germany

## Abstract

Tripartite multidrug efflux systems of Gram-negative bacteria are composed of an inner membrane transporter, an outer membrane channel and a periplasmic adaptor protein. They are assumed to form ducts inside the periplasm facilitating drug exit across the outer membrane. Here we present the reconstitution of native *Pseudomonas aeruginosa* MexAB–OprM and *Escherichia coli* AcrAB–TolC tripartite Resistance Nodulation and cell Division (RND) efflux systems in a lipid nanodisc system. Single-particle analysis by electron microscopy reveals the inner and outer membrane protein components linked together via the periplasmic adaptor protein. This intrinsic ability of the native components to self-assemble also leads to the formation of a stable interspecies AcrA–MexB–TolC complex suggesting a common mechanism of tripartite assembly. Projection structures of all three complexes emphasize the role of the periplasmic adaptor protein as part of the exit duct with no physical interaction between the inner and outer membrane components.

## Abstract (web summary)

Tripartite efflux systems consist of inner membrane, outer membrane and periplasmic components. Here, Daury *et al*. reconstitute native versions of RND transporters in nanodiscs and present projection structures emphasizing the role of the periplasmic adaptor in linking the inner and outer membrane proteins.

## Graphical abstract

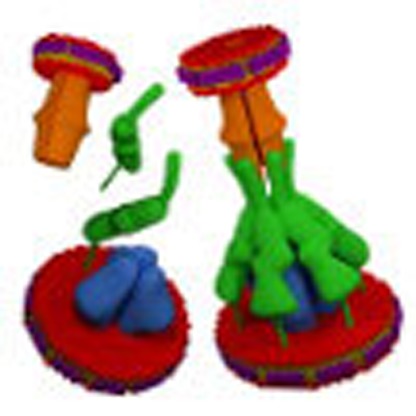

Gram-negative efflux pumps of the Resistance Nodulation cell Division (RND) superfamily are exporters of biological metabolites and antimicrobial compounds, thus playing a prominent role in the bacterial resistance, which has nowadays become a major health concern^1^,^2^. The inner membrane located RND pumps are driven by the proton motive force and as a part of a tripartite system, working in conjunction with an outer membrane factor (OMF), and a periplasmic membrane fusion protein (MFP), the latter assumed to link the RND component to the OMF^3^. Well-studied examples of tripartite RND systems are MexAB–OprM of *Pseudomonas aeruginosa* and AcrAB–TolC of *Escherichia coli*^3^. For these systems, high-resolution X-ray structures are available for the single components TolC^4^, AcrA^5^, AcrB^6^,^7^,^8^,^9^,^10^, OprM^11^,^12^, MexA^13^,^14^ and MexB^15^. OprM/TolC possesses a trimeric organization consisting in a 4-nm-long transmembrane domain comprising 12 strands that form a β-barrel and a 10-nm-long periplasmic domain comprising 12 α-helices and a mixed α/β equatorial domain^11^. MexB/AcrB forms a trimer in which each protomer is made of a 12 transmembrane α-helices domain and a large periplasmic part comprising a porter and a funnel domain extending 7 nm away from the inner membrane inside the periplasm^6^,^7^,^8^,^9^,^15^. MexA/AcrA is arranged in four consecutive domains, that is, membrane proximal, β-barrel, lipoyl and α-helical hairpin domains. MexA/AcrA has been shown to be anchored to the inner membrane via palmitoylation of an N-terminal cysteinyl residue^13^,^14^,^16^. It has been postulated that drugs are transported from the periplasmic side across the outer membrane in an energy-dependent manner via the RND protein and the OMF channel^7^,^8^,^9^,^15^,^16^,^17^,^18^,^19^,^20^,^21^. This intriguing transport mechanism is suggested to occur via a peristaltic mode through the protomers of the trimeric RND component caused by consecutive functional cycling of the protomers through three different states (loose, tight and open or access, binding and extrusion)^8^,^9^,^10^,^20^,^22^,^23^,^24^. Despite these structural and computational insights, only a few inhibitors of these efflux pumps have been described thus far^22^,^25^,^26^,^27^,^28^. These compounds function as competitive inhibitors or impair the proper binding of substrates and are in one case^25^ also transported by the efflux pump system, albeit at a very low rate. To embark on the development of allosteric inhibitors, for example, those preventing the tripartite setup, the assembly of the tripartite system itself has to be understood.

This is particularly challenging, since these systems span two different membranes and the periplasm of the Gram-negative cell, hence studies related to the assembly mechanism face many methodological difficulties. In the search of understanding the assembly mechanism, bipartite MFP–RND and MFP–OMF complexes have been reconstituted *in vitro*. AcrA–AcrB and AcrA–TolC interactions have been confirmed with detergent-solubilized proteins^29^,^30^,^31^. Recently a crystal structure of the heavy-metal CusBA transporter revealed six MFP proteins interacting with a RND trimer^32^. In addition, the architecture of bipartite OprM–MexA complexes sandwiched between two lipid membranes studied by cryo-electron tomography revealed a 21-nm intermembrane distance^33^. Evidence for a direct interaction between RND and OMF relied on *in vitro* AcrB–TolC binding^30^ and *in vivo* cross-linking studies^34^,^35^ suggesting a limited interface between these two membrane proteins^16^.

To date, there are only few studies reporting on the assembly of the tripartite complex. In 2011, AcrAB–TolC assembly immobilized on a surface has been monitored by plasmon resonance surface^30^. And very recently, single-particle electron microscopy (EM) models, in one case including a fourth partner^36^, AcrZ^37^, have been described, where the detergent-solubilized tripartite AcrAB–TolC^38^ or tetrapartite AcrABZ–TolC^36^ setup was stabilized by genetic fusion constructs of the complex components (and chemical cross-linkers^36^).

Here we report the reconstitution of native MexAB–OprM, AcrAB–TolC and interspecies AcrA–MexB–TolC complexes using nanodisc (ND) technology^39^. The visualization by single-particle EM reveals tripartite complexes made of the inner and outer membrane protein components linked together via the periplasmic adaptor protein emphasizing its role as part of the exit duct with no physical interaction between the inner and outer membrane components.

## Results

### Protocol of tripartite assembly using NDs

**Figure 1:**
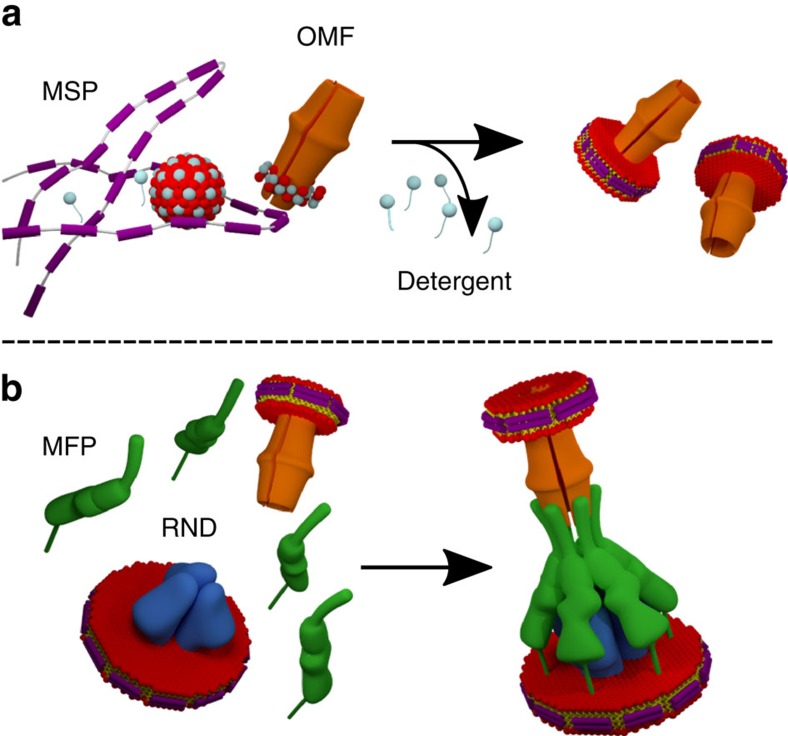
Tripartite assembly based on RND and OMF inserted into nanodiscs. (**a**) After detergent removal, the integral membrane proteins are reconstituted into a small lipid bilayer wrapped by two MSPs (purple) forming the nanodisc. Lipids are red/yellow and detergent is grey. (**b**) Self-assembly of RND (blue) and OMF (orange) in nanodiscs in the presence of native lipid-modified MFP (green) leading to the tripartite complex in lipid membrane.

The rationale for the reconstitution of tripartite complexes was based on the insertion of the integral membrane proteins (that is, OMF or RND) into NDs. On detergent removal, the membrane proteins (MexB, AcrB, OprM and TolC) were inserted into a 1-palmitoyl-2-oleoyl-*sn*-glycero-3-phosphocholine (POPC)-containing NDs whose size is limited by the membrane scaffold protein (MSP)^40^ wrapped around the hydrophobic core of the lipids (Fig. 1a). The control of the assembly process relied on the insertion of a single molecule per ND, which necessitated the use of two MSP differing in size (MSP1D1 or MSP1E3D1) because of the respective diameters of the transmembrane domains of the RND and OMF proteins (RND≈80 Å, OMF≈40–55 Å (refs 7, 11)). Subsequently, the separately ND-reconstituted efflux components were mixed with native lipidated MFP (AcrA or MexA, Fig. 1b).

This two-step reconstitution protocol was successfully applied to MexAB–OprM and AcrAB–TolC. In the following sections, the reconstitution is detailed by first characterizing OprM or MexB into ND, followed by the process of whole-tripartite assembly reconstitution. Moreover, reconstitution of cognate AcrAB–TolC and non-cognate AcrA–MexB–TolC complexes are presented highlighting the generic approach of our protocol. The ND-reconstituted native tripartite complexes were visualized by EM and single-particle analysis, resulting in elongated structures of 33 nm along their main axis.

### OprM and MexB molecules inserted into NDs

**Figure 2:**
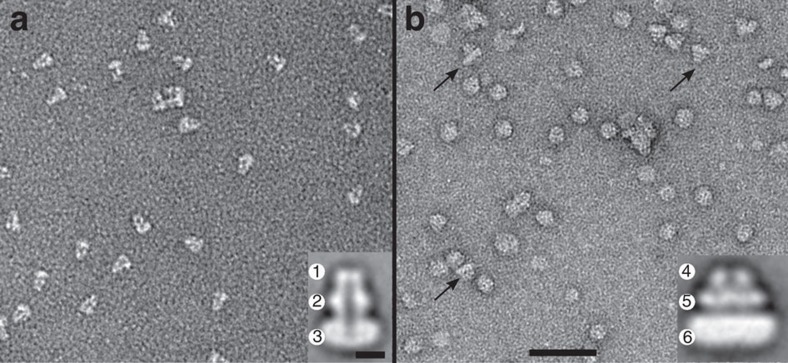
TEM observations of OprM and MexB reconstituted into nanodiscs. (**a**) Field of view of OprM–ND showing side views of isolated molecules. The average image (inset) reveals characteristic features: The OprM β-barrel in the ND (1) and the OprM periplasmic domain composed of the equatorial domain (2) and the tip of the α-barrel (3) protruding from the ND. (**b**) Field of view of MexB-ND showing isolated molecules. Black arrows indicate side views. On the average image (inset), a side view of MexB exhibits the periplasmic part organized in two layers (funnel (4) and porter (5) domains) protruding from the ND (6). Scale bars, 50 nm and 5 nm for the inset. a.u., arbitrary unit.

The ND reconstitution of OprM was achieved with construct MSP1D1 and POPC lipids reported to form 10-nm-diameter sized NDs. Analysis of these OprM–NDs by EM was done on negatively uranyl acetate-stained samples. At a MSP:lipid:OprM molar ratio of 1:36:0.4, OprM–ND mainly contained one OprM molecule per ND, with their long axis preferentially oriented parallel to the carbon support (Fig. 2a). EM observations were consistent with the trimeric assembly of OprM^41^. An average image (from 446 particles) of the OprM–ND revealed a 11 nm in diameter ND spanned by a duct formed by the OprM β-barrel domain (visible due to the presence of uranyl acetate within the β-barrel), followed by the 10-nm-long OprM periplasmic domain including the equatorial domain (Fig. 2a inset). Note that MSP1E3D1 construct produced larger ND leading to the insertion of two OprM molecules (Supplementary Fig. 1).

The formation of MexB–ND was achieved using POPC and MSP1E3D1 as scaffold resulting in 12- to 14-nm-diameter sized NDs (that is, a diameter larger than the transmembrane domain of MexB). At a MSP:lipid:MexB molar ratio of 1:27:1, EM revealed side views of MexB–ND containing one molecule per ND (Fig. 2b) in accordance with the trimeric organization of MexB (Protein Data Bank entry: 2V50). Clearly visible is also the exposed periplasmic domain that protrudes 7 nm away from the lipid-containing ND. Averaging 341 single particles revealed a continuous layer of electron density of the ND, including the 36-transmembrane helix domain of trimeric MexB. The periplasmic part of MexB exhibited furthermore two clearly distinguishable layers of density, assigned to the porter domain and to the more distal funnel domain (Fig. 2b inset).

### Formation of a tripartite complex

**Figure 3:**
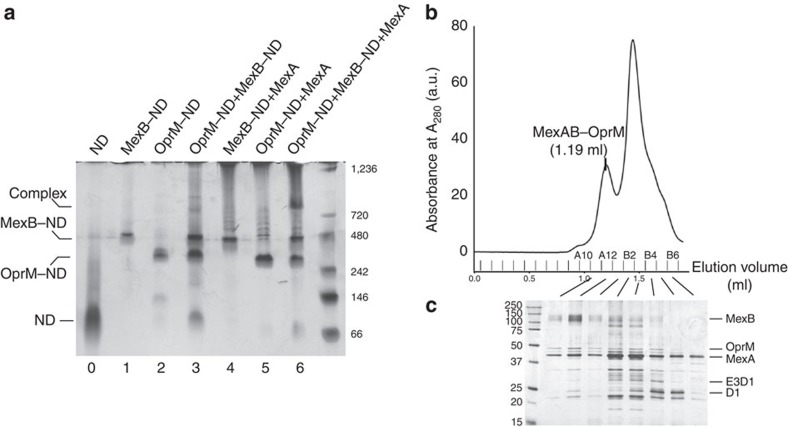
Native PAGE analysis and purification of the tripartite MexAB–OprM assembly. (**a**) Electrophoretic mobility shift assay of individual and mixed components. OprM–ND and MexB–ND were mixed in the presence and in the absence of MexA. An extra band was observed when the three components were present in the sample. Lane 0, ND; lane 1, MexB–ND; lane 2, OprM–ND; lane 3, MexB–ND and OprM–ND; lane 4, MexB–ND and MexA; lane 5, OprM–ND and MexA; lane 6, MexB–ND, OprM–ND, and MexA. Proteins were separated by native PAGE and stained with PlusOne Silver Staining Kit. (**b**) Analytical size-exclusion chromatography (SEC) analysis of the mixed components. (**c**) SDS–PAGE analysis of the indicated SEC peak fractions. The molecular mass of each marker protein (in kilodalton) is indicated on the right (**a**) and on the left (**b**).

Tripartite complex formation was achieved by mixing OprM–ND, MexB–ND and lipidated MexA in a 1:1:10 molar ratio (Fig. 1b). Formation of tripartite complexes was visualized using native PAGE resulting in an electrophoretic mobility shift on complex formation (Fig. 3a). ND-reconstituted MexB and OprM migrated as a single band strongly stained by silver (Fig. 3a, lanes 1 and 2). MexB–ND migration was less than OprM–ND due to its larger size and hydrodynamic radius. Mixing of MexB–ND with OprM–ND in a 1:1 molar ratio yielded two separate stained bands (Fig. 3a, lane 3) with corresponding electrophoretic mobilities of the two single ND-reconstituted components (Fig. 3a, lanes 1 and 2). However, in a mixture containing MexB–ND, OprM–ND and lipidated MexA (39 kDa monomer; 1:1:10 molar ratio), a significant upshifted band was observed after staining with silver (Fig. 3a, lane 6). This extra band was only observed when all three components were mixed. When lipidated MexA was mixed with either MexB–ND (Fig. 3a, lane 4) or with OprM–ND (Fig. 3a, lane 5), no upshift of bands could be observed. Our interpretation is that MexB–ND and OprM–ND do not form bipartite complexes and only when the three components of the tripartite complex (that is, MexB–ND, OprM–ND and lipidated MexA) are present in the sample, a larger complex is formed.

**Figure 4:**
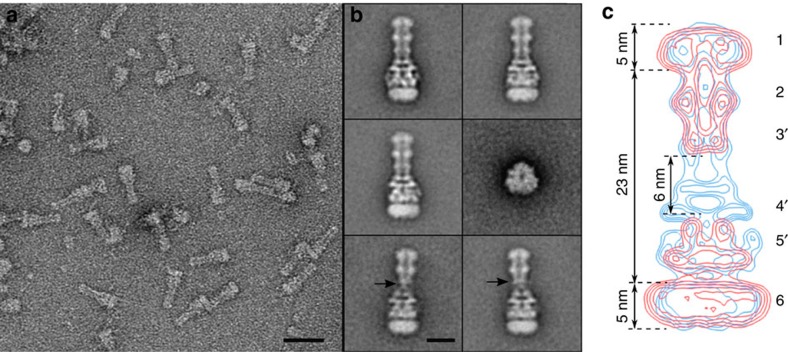
TEM analysis of tripartite MexAB–OprM assembly. (**a**) Field of view revealing elongated complexes when OprM-ND and MexB-ND were mixed in the presence of MexA. Scale bar, 30 nm. (**b**) Gallery of five class average side views of a 33-nm-long tripartite MexAB–OprM complex delineated by two nanodiscs (157, 192, 185, 99, 151 images, respectively) and one top view class average (56 images). Lower row, two average classes of atypical complexes showing faint contacts between OprM and MexAB (black arrows). Scale bar, 10 nm. (**c**) Isocontours of MexB–ND and OprM–ND (red) overlaid on isocontours of tripartite complex (blue). Characteristic features are displayed: OprM β-barrel and ND (1); equatorial domain (2), tip of α barrel (3′), MexB funnel (4′) and porter (5′) domains anchored to ND (6). The remaining blue densities correspond to MexA that linked OprM to MexB and interacts with the domains marked with (′). The tripartite assembly ND (6) has a smaller size compared with ND of MexB probably because of the detergent carried with MexA that may extract some lipids.

To get structural details on the assembly of the efflux pump, the (1:1:10) mixture of OprM–ND, MexB–ND and lipidated MexA was analysed by EM (Supplementary Fig. 2). Strikingly, within the population of single particles, *ca.* 10% were elongated structures of 33 nm at their longest expansion. Clearly different from isolated MexB–ND and OprM–ND (Fig. 2a,b), these new structures likely correspond to complex formation evidenced on the silver-stained native polyacrylamide gel (Fig. 3a, lane 6). To improve the yield of tripartite complex, the assembly formed with OprM–ND, MexB–ND and lipidated MexA in a 1:1:20 molar ratio was purified by size-exclusion chromatography (SEC) and analysis of the fractions by SDS–PAGE (Fig. 3c) revealed the presence of the three partners, in particular, in the first peak (Fig. 3b, fraction A12). Note that OprM–ND and MexB–ND were eluted in B4 and B3 fractions, respectively when applied alone on the same column (Supplementary Fig. 3). EM analysis of A12 fraction exhibited a vast majority of elongated structures viewed from their sides (Fig. 4a). The majority of class averages (125 over 200 classes) from single-particle average image analysis revealed an edifice of protein densities at both ends resembling ND densities ∼23 nm apart (Fig. 4b). The upper part of the complex resembled OprM–ND with its central duct, including the ND-surrounded β-barrel domain. Adjacent to the ND density, a bulky knot is visible, which we interpret as the equatorial domain (Fig. 4b,c). At the other end of the elongated particle, we observe the recognizable features of MexB, that is, the ND-embedded transmembrane domain, and the protruding MexB porter and funnel domains (Fig. 4b,c). In between the OprM–ND and MexB–ND densities, additional densities are present, which we assign to connecting MexA molecules. Isocontours of OprM–ND and MexB–ND (derived from the average images of Fig. 2), overlaid on the isocontours of the putative tripartite complex average image, showed a good match of densities of ND-embedded OprM and MexB molecules (Fig. 4c). The 6-nm long, non-overlapping densities most likely corresponded to MexA molecules that are interacting with both OprM and MexB (blue contours in Fig. 4c). Hence, in accordance with this analysis, it can be concluded that the tripartite MexB–MexA–OprM complex was successfully formed using NDs reconstitution. Comparison of the intermembrane distance (23 nm, Fig. 4c) with the known dimensions of the periplasmic domains of MexB (7 nm (ref. 15)) and OprM (10 nm (refs 11, 12)) and the observed non-overlapping density of ∼6 nm suggest that the RND (MexB) and OMF (OprM) components are not in direct contact. This observation is in accordance with the results recently obtained with genetically fused RND–MFP constructs^36^,^38^, supporting the role of MexA as a part of the periplasmic duct formed by the MexB–MexA–OprM complex, bridging the gap between MexB and OprM (Supplementary Fig. 4).

Interestingly, few class averages (about 400 particles) showed that the contact between MexA and OprM are apparently different. OprM appears to contact the MexAB complex over MexA via small links (black arrows Fig. 4b). These extremities arising from the α-barrel of OprM and engaged the interaction with MexA resemble those observed in images of isolated OprM, which in an isolated state is present in a closed α-barrel conformation^11^ (Fig. 2). The complexes showing the closed OprM engaged in a presumably looser manner with the MexAB complex may correspond to intermediate steps in the formation of the tripartite system, even though we cannot exclude that these particles represent dissociated complexes due to their interaction on the EM grid.

### Tripartite complex of AcrAB–TolC in NDs

**Figure 5:**
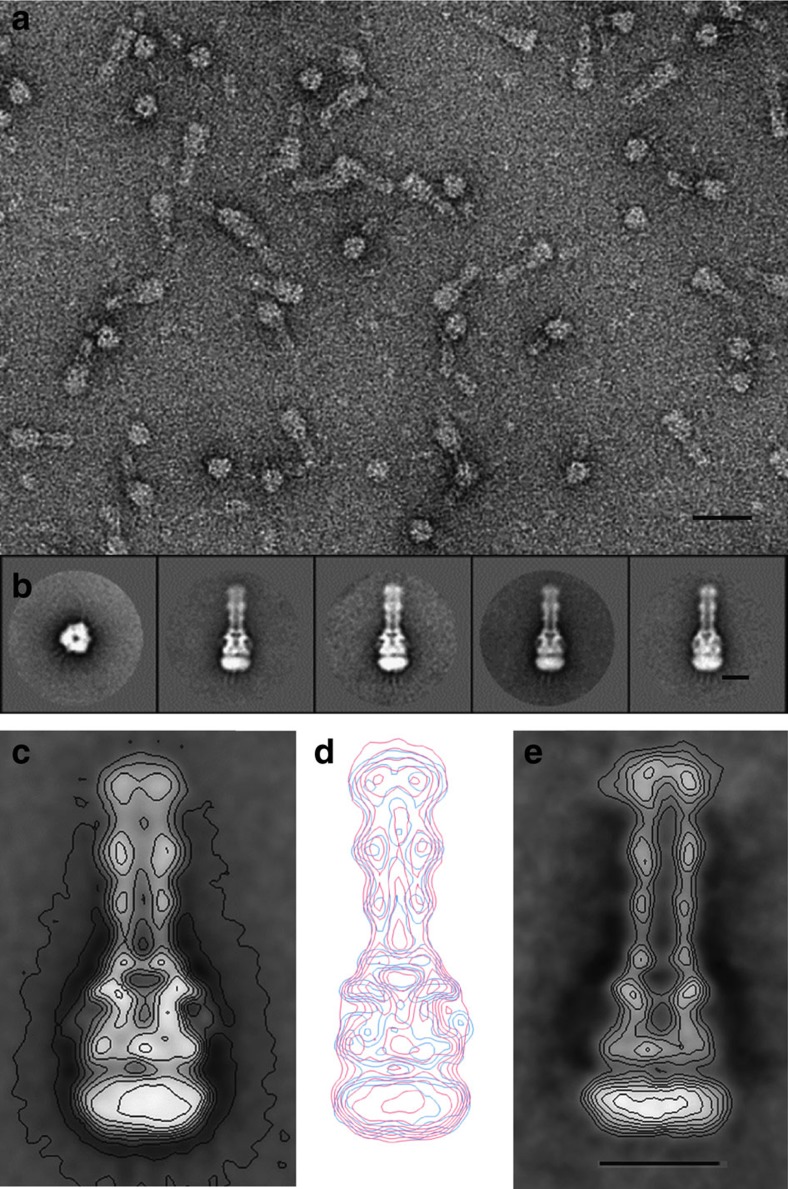
TEM analysis of tripartite AcrAB–TolC and AcrA–MexB–TolC assemblies. (**a**) Field of view revealing purified AcrAB–TolC assemblies. Scale bar, 30 nm. (**b**) Gallery of one top view class average (137 images) and four class average side views of a 33-nm-long tripartite AcrAB–TolC complex (223, 119, 196, 178 images, respectively) and. (**c**) Enlarged average image of tripartite AcrAB–TolC assembly. (**d**) Isocontours of tripartite AcrAB–TolC assembly (red) overlaid on isocontours of tripartite MexAB–OprM assembly (blue). (**e**) An average of tripartite AcrA–MexB–TolC assembly. Scale bar, 10 nm for **c**,**d**,**e**.

A similar reconstitution procedure was applied to the AcrB–ND, TolC–ND and lipidated AcrA. An average image of TolC side views (average over 444 images) revealed elongated protein densities protruding from the ND and forming a tunnel/duct similar to that observed for OprM (Supplementary Fig. 5a). Side views of AcrB molecules inserted into NDs (average over 294 images) showed two main layers of protein densities resembling those of MexB molecules, that is, ND/transmembrane, porter and funnel domains (Supplementary Fig. 5b). After mixing TolC–ND, AcrB–ND and AcrA in a 1:1:10 ratio, gel filtration was performed to purify the tripartite complex for further EM analysis (Fig. 5a and Supplementary Fig. 6). Average classes of side and top views showed that a complex was composed of TolC–ND (upper part), AcrB–ND (lower part) and connecting those, densities assigned to AcrA molecules were observed in the negative-stained averaged image (Fig. 5b,c). Interestingly, the intermembrane/ND distance was similar to that of MexAB–OprM tripartite complex, likewise suggesting that there is no direct contact between TolC and AcrB (Fig. 5d). Clearly visible from this comparison, the tripartite assemblies of AcrAB–TolC and of MexAB–OprM share similarities suggesting a common mechanism for tripartite assembly.

### Hybrid tripartite complex of AcrA–MexB–TolC in NDs

Thus far, we used cognate components for our analysis of tripartite assembly. Since the image analysis suggested a common mechanism of tripartite formation and complex assembly, we also analysed mixtures of non-cognate TolC–ND, MexB–ND and AcrA as well as OprM–ND, AcrB–ND and MexA (1:1:10 ratio) by EM. Surprisingly, for the MexB–AcrA–TolC mixture, we also encountered complexes albeit less frequent (236 complexes), with an overall appearance similar to the cognate tripartite complexes (Fig. 5e and Supplementary Fig. 7). Even for the non-cognate mixture of OprM–ND, AcrB–ND and MexA tripartite complex particles were observed, but at a very low frequency (<1%). The formation of a lower amount of hybrid (non-cognate) complexes compared with genuine ones suggests that the components most likely exhibit much lower binding affinities.

## Discussion

We have devised a generic approach allowing the formation of tripartite RND efflux pumps as characterized by native gel and EM analysis. By means of a ND toolkit, integral membrane components of two tripartite RND efflux systems were incorporated into a lipid membrane ND and were further assembled after adding purified lipidated MFPs. Similar tripartite assemblies were obtained for the two different pumps from *E. coli* and from *P. aeruginosa,* that is, AcrB–AcrA–TolC and MexB–MexA–OprM, respectively. With an overall height of 33 nm, the tripartite complexes were composed of an OMF molecule and an RND molecule facing each other with their periplasmic domains without being in direct contact, linked by MFP molecules. These self-assembled tripartite complexes strongly resembled the AcrABZ–TolC edifice obtained using protein fusion^36^ and suggests a 3:6:3 stoichiometric assembly of the components. In this report, the tripartite assemblies comprised native proteins and self-assembly of the three components occurred without any additional protein(s) or chemical cross-linking. Interestingly, both tripartite setups based on native (this work) or fused^36^,^38^ components indicated a common organization of the MFP connecting OMF and RND components at this level of detail (Supplementary Fig. 4). The three-dimensional reconstruction of MexAB–OprM (Supplementary Fig. 4a) at an estimated resolution of 25 Å (Supplementary Fig. 4b) displays the distinct features of the tripartite system (that is, OMF, MFP-hairpin, -lipoyl, -β-barrel and -membrane proximal domains, in addition to the RND transporter), which is in good agreement with the cryo-EM structure by Du *et al*.^36^ (Supplementary Fig. 4c). This organizational resemblance also suggests that our tripartite pump systems were assembled with similar stoichiometry, that is, 3:6:3 as has been previously proposed for other tripartite pumps as well^32^,^42^. It is worth noting that by mixing MexB–ND and OprM–ND, complex formation was not observed on native gel or on EM grid suggesting that OprM does not strongly bind to MexB in solution as was previously observed by isothermal titration calorimetry for the homologous partners TolC and AcrB^31^.

Our results also provided evidence that RND tripartite systems were able to self-assemble in a mixture of non-cognate components. When MexB was interchanged with AcrB in the self-assembling process, tripartite AcrA–MexB–TolC complexes were observed but with a lower occurrence compared with the formation of cognate complexes. The formation of hybrid tripartite complexes has a significant meaning since it has been observed that AcrA–MexB–TolC hybrid system could confer resistance to *E. coli* for only a limited subset of drugs normally transported by the cognate efflux systems^43^. The fact that we observed only few hybrid AcrA–MexB–TolC complexes may also explain the partial resistance observed in an *E. coli* Δ*acrB* background complemented with heterologous expressed *mexB*. MexA or AcrA are known for their high flexibility most likely facilitating tripartite formation^44^. The spectrum of antibiotic resistance of the non-cognate efflux systems could be extended by side chain substitutions in the helical hairpin of AcrA, which interacts with TolC or by substitutions in the AcrA β-barrel domain interacting with MexB^43^. The use of variants of AcrA^43^, TolC^45^ or MexB^46^ may provide a better fit with the two other components. Since similar tripartite assemblies were formed with cognate and non-cognate components, as schematically shown in Fig. 5e, we suggest that there might be a general mechanism triggering tripartite assembly, the formation efficiency being dependent on the binding affinities of the MFPs, therefore modulating drug efflux.

As discussed above, the MFP component possesses a high adaptability in the maintenance of interactions between the OMF and RND components. It has been proposed that during drug export, conformational changes of the MFP component, triggered by substrate and/or H^+^ binding and/or release to/from the RND component could catalyse the assembly/disassembly of the tripartite complex^47^. The role of the MFP as a central element with conformational change capability might indicate that the tripartite assembly could be a multi-stage process. The observed assemblies in this study and in Du and Luisi's work^36^ might represent the final stage formation of a fully functional tripartite setup. However, we cannot exclude that the observed setup might be an intermediate formation that, under *in vivo* conditions, is subsequently subjected to a yet unknown conformational step leading towards fully functional tripartite complex, where the OMF and RND components are in direct contact. This might explain the previously reported direct cysteine disulfide cross-linking between AcrB and TolC^34^ and would possibly indicate an energy requirement (for example, substrate-binding energy and/or proton motive force) for functional tripartite complex formation.

The reconstitution of the tripartite assembly opens new perspectives for the development of efflux pump inhibitors (EPIs), precluding antibiotic efflux and resulting in an increase of intracellular antibiotic concentration. Efforts have been concentrated up till now on the search for compounds that could compete for antibiotic-binding sites and/or block drug translocation^48^,^49^. Nevertheless, such inhibitors are particularly difficult to identify owing to the broad variety of substrates that the multidrug pumps can accommodate. Another strategy would be to target the assembly of the tripartite system. The generation of a leak in the channel by preventing the protein/protein recognitions or by hampering their structural adaptability could also affect the pump efficiency. Compounds having such effect would represent a class of EPIs acting on the assembly process of efflux pumps, instead of acting on drug translocation. Interestingly, unlike RND proteins, OMFs have often been observed to be shared among different tripartite systems, independent of the nature of the inner membrane protein or periplasmic adaptor component^50^. In the case of *P. aeruginosa*, OprM can operate with MexAB, MexXY^51^ and MexMN^52^. An inhibitor compound precluding OprM–MexA interaction could therefore also inhibit the activity of the other pumps.

Our protocol opens the way towards a better understanding of the tripartite assembly of AcrAB–TolC and MexAB–OprM. RND and OMF components inserted into NDs are able to self-assemble in a tripartite complex in the presence of a lipidated MFP component. We provide evidence for a common mechanism of tripartite assembly with cognate and non-cognate components, whereby MFP molecules link their cognate OMF partners to cognate and non-cognate RND components. From the single-particle analysis, there is clear evidence that the RND and OMF components are not in direct contact within the tripartite assembly. This approach can be extended to a vast number of RND pump systems and open the field for further structural analysis at atomic level. In addition, the development of an EPI class targeting the tripartite assembly process can be approached more systematically. Although our protocol is not suitable for a large screening of compounds, it is a proof of concept for inhibitor compound analysis.

## Methods

### Materials and reagents

POPC was purchased from Avanti Polar Lipids (USA), sodium cholate hydrate, octyl-poly-oxyethylene, n-octyl-β-D-glucopyranoside (OG) and *n*-Dodecyl β-D-maltoside (DDM) were purchased from Sigma. SM2 Bio-beads and 4–15% Mini-PROTEAN TGX gel were obtained from Bio-Rad. Superose 6 3.2/300 column and PlusOne Silver Staining Kit were purchased from GE Healthcare. Cu 300 mesh grids were obtained from Agar Scientific.

### Lipid preparation

POPC lipids were dissolved in methanol/chloroform (v/v), dried onto a glass tube under steady flow of nitrogen and followed by exposure to vacuum for 1 h. The lipid film was suspended in the reconstitution buffer (100 mM NaCl, 10 mM Tris/Cl, pH 7.4) and subjected to five rounds of sonication for 30 s each. Lipid concentration was quantified by phosphate analysis.

### Protein preparation

Two MSPs, MSP1D1 and MSP1E3D1 (genetic constructs available from AddGene) expressed and purified from bacteria^40^, were used to make OprM/TolC and MexB/AcrB NDs, respectively. The *acrA* and *tolC* genes were individually cloned into pET24a after amplification with primers acrA_forward 5′-GATTCGGGGCCCAACAAAAACAGAGGGTTTACG-3′, acrA_reverse 5′-ATAATAGGATCCTTAAGACTTGGACTGTTCAGGCTG-3′, tolC_forward 5′-AAAACATATGAAGAAATTGCTCCCC-3′ and tolC_reverse 5′-AAAACTCGAGGTGGTGGTGGTGGTTACGGAAAGGGTTATGACCGTT-3′ containing *Nde*I and *Xho*I restriction sites, analogous to the cloning of *acrB* as described previously^53^. AcrA, AcrB and TolC were separately produced in *E. coli* C43(DE3)Δ*acrAB* and subsequently purified as previously described for AcrB^53^. In brief, cells were grown in TB medium at 37 °C at 180 r.p.m. till OD_600_ of 1.3 before induction with 0.5 mM IPTG. After induction, the cells were grown overnight at 20 °C at 180 r.p.m. After harvest, cells were suspended at 5 g cells wet weight per ml in lysis buffer (Tris/Cl 20 mM pH 7.5, NaCl 500 mM, MgCl_2_ 2 mM, 100 μM PefaBloc (Sigma), and trace amounts of DNAse I and lysozyme). Cells were lysed by three passages through a Constant Systems cell disruptor at 22 kpsi and centrifuged for 30 min at 18,000*g* at 4 °C. The supernatant was subsequently centrifuged for 1 h at 180,000*g* at 4 °C to collect the membranes. Membranes were solubilized (10 ml per g wet weight membranes) in lysis buffer containing 10 mM imidazole, pH 7.5, and 2% DDM for 1 h at 4 °C while gently stirring. Samples were centrifuged for 1 h at 180,000*g* and the supernatant was subjected to Ni-NTA affinity chromatography. The proteins were eluted in a 10 ml buffer (Tris/Cl 20 mM, pH 7.5, NaCl 150 mM, imidazole 200 mM pH 8.0, and 0.03% DDM). AcrA and AcrB were then subjected to SEC using a Superpose 6 HR column (GE Healthcare) with Tris/Cl 20 mM pH 7.5, NaCl 150 mM and 0.05% DDM as running buffer. The TolC sample was passed through a desalting column (NAP-10, GE Healthcare) using Tris/Cl 20 mM pH 7.5, NaCl 150 mM and 1.5% OG as buffer.

MexB, MexA and OprM membrane proteins were separately produced in *E. coli* C43Δ*acrB* and purified as described for MexB^54^, MexA^13^ and OprM^12^,^55^. In brief, cells were grown in TY medium at 37 °C at 200 r.p.m. till OD_600_ of 0.6 before induction with 1 mg ml^−1^ IPTG. After induction, the cells were grown overnight at 20 °C at 200 r.p.m. After harvest, cells were suspended in lysis buffer (Tris/Cl 20 mM pH 8, NaCl 200 mM). Cells were lysed by two passages through a Constant Systems cell disruptor at 35 kpsi and centrifuged at 10,000*g* for 25 min at 4 °C. For MexB and MexA, the supernatants were submitted to an ultracentrifugation at 100,000*g* for 1 h at 4 °C. For OprM, the supernatant is solubilized with 2% n-octyl-poly-oxyethylene before ultracentrifugation to get rid of the inner membranes. Membranes were subsequently submitted to an overnight detergent solubilization in Bis-Tris 10 mM pH 7.4, glycerol 20%, imidazole 10 mM and NaCl 500 mM and at a 2:1 and 40:1 (w/w) detergent-to-protein ratio for MexB and MexA/OprM, respectively (protein concentration is determined using the Bicinchoninic acid test from Sigma). Samples were subjected to an ultracentrifugation at 100,000*g* for 1 h at 4 °C. Protein purifications were performed by affinity chromatography on a HisTrap HP column followed by a gel filtration (superose 6 HR, 10/300 GE Healthcare). After purification, protein buffers contained 0.03% DDM for MexA and MexB and 0.9% OG for OprM.

### Preparation of MexB/AcrB and OprM/TolC in NDs

MexB and OprM were inserted into NDs according to the standard protocol^39^,^40^. Briefly, to obtain MexB–ND, MexB solution was mixed with POPC solution and MSP solution at a final 27:1:1 lipids/MSP/protein molar ratio in a 10 mM Tris/Cl, pH 7.4, 100 mM NaCl with 0.009% DDM and 15 mM Na-cholate solution. Detergent was removed by the addition of SM2 Bio-beads into the mixture shaken overnight at 4 °C. For OprM–ND, OprM solution was mixed with POPC solution and MSP solution at a final 36:1:0.4 lipids/MSP/protein molar ratio in a 10 mM Tris/Cl, pH 7.4, 100 mM NaCl, 30 mM OG and 15 mM Na-cholate solution. Like for MexB–ND, detergent was removed with SM2 Bio-beads overnight at 4 °C. The same protocol was used for AcrB and TolC proteins.

### Purification of the tripartite complex

To enrich the tripartite complexes from the initial mixed samples, we subjected the MexAB–OprM (or AcrAB–TolC) containing samples to SEC using a Superose 6 column pre-equilibrated with reconstitution buffer, which was also used as running buffer. Peak fractions were collected and subjected to SDS–PAGE analysis. The resulting gel was stained with PlusOne Silver Staining Kit.

### Native gel analysis

For native gel electrophoresis, samples (0.5 to 2 μl) were separated on 4–15% continuous gradient polyacrylamide Mini-PROTEAN TGX gel in sample buffer (500 mM Tris/Cl pH 6.8, 30% glycerol, 0.05% bromophenol blue). Electrophoresis was performed at constant voltage of 150 V for 90 min, in Tris-Glycine running buffer. The gel was stained with PlusOne Silver Staining Kit.

### Electron microscopy acquisition and analysis

For EM grid preparations, a diluted mixture of the samples suspension was applied to a glow-discharged carbon-coated copper 300 mesh grids and stained with 2% uranyl acetate (w/v) solution. Images were recorded under low-dose conditions on electron microscope (Tecnai 12 and F20 FEG, FEI) using a FEI Eagle 4k × 4k and a Gatan USC1000 2k × 2k cameras. Image alignment and two-dimensional averages were performed with SPIDER using a reference-free alignment procedure. For MexAB–OprM and for AcrAB–TolC, a total of 22,979 and 28,863 particles, respectively, were automatically selected and processed for class averaging.

For assessing the occurrence of complex formation, OprM–ND, MexB–ND and lipidated MexA were mixed at a ratio of 1:1:10, and sampled after 1, 6, 12, 24, 48, 96 and 168 h (1 week) and from there on every 168 h up to 6 weeks. For each sample, two negatively stained grids were prepared and subjected to transmission electron microscope. Twenty-five micrographs were randomly collected per grid (total 50 micrographs per sample). The occurrence of the complexes was expressed as a ratio of complexes present on the grid corresponding to the number of complexes determined by a manual picking divided by the number of objects (ND alone; MexB–ND, OprM–ND and complexes) calculated by automatic picking based on the finding of maximum values in a Difference of Gaussian filtered micrograph.

## Additional information

**How to cite this article**: Daury, L. *et al*. Tripartite assembly of RND multidrug efflux pumps. *Nat. Commun.* 7:10731 doi: 10.1038/ncomms10731 (2016).

## Supplementary Material

Supplementary Information Supplementary Figures 1-7

## References

[b1] May M. Drug development: time for teamwork. Nature 509, S4–S5 (2014). 24784427 10.1038/509S4a

[b2] Li X.-Z., Plésiat P. & Nikaido H. The challenge of efflux-mediated antibiotic resistance in Gram-negative bacteria. Clin. Microbiol. Rev. 28, 337–418 (2015). 25788514 10.1128/CMR.00117-14 PMC4402952

[b3] Nikaido H. Multidrug resistance in bacteria. Annu. Rev. Biochem. 78, 119–146 (2009). 19231985 10.1146/annurev.biochem.78.082907.145923 PMC2839888

[b4] Koronakis V., Sharff A., Koronakis E., Luisi B. & Hughes C. Crystal structure of the bacterial membrane protein TolC central to multidrug efflux and protein export. Nature 405, 914–919 (2000). 10879525 10.1038/35016007

[b5] Mikolosko J., Bobyk K., Zgurskaya H. I. & Ghosh P. Conformational flexibility in the multidrug efflux system protein AcrA. Structure 14, 577–587 (2006). 16531241 10.1016/j.str.2005.11.015 PMC1997295

[b6] Eicher T. . Transport of drugs by the multidrug transporter AcrB involves an access and a deep binding pocket that are separated by a switch-loop. Proc. Natl Acad. Sci. USA 109, 5687–5692 (2012). 22451937 10.1073/pnas.1114944109 PMC3326505

[b7] Murakami S., Nakashima R., Yamashita E. & Yamaguchi A. Crystal structure of bacterial multidrug efflux transporter AcrB. Nature 419, 587–593 (2002). 12374972 10.1038/nature01050

[b8] Murakami S., Nakashima R., Yamashita E., Matsumoto T. & Yamaguchi A. Crystal structures of a multidrug transporter reveal a functionally rotating mechanism. Nature 443, 173–179 (2006). 16915237 10.1038/nature05076

[b9] Seeger M. A. . Structural asymmetry of AcrB trimer suggests a peristaltic pump mechanism. Science 313, 1295–1298 (2006). 16946072 10.1126/science.1131542

[b10] Sennhauser G., Amstutz P., Briand C., Storchenegger O. & Grütter M. G. Drug export pathway of multidrug exporter AcrB revealed by DARPin inhibitors. PLoS Biol. 5, e7 (2007). 17194213 10.1371/journal.pbio.0050007 PMC1717020

[b11] Akama H. . Crystal structure of the drug discharge outer membrane protein, OprM, of Pseudomonas aeruginosa: dual modes of membrane anchoring and occluded cavity end. J. Biol. Chem. 279, 52816–52819 (2004). 15507433 10.1074/jbc.C400445200

[b12] Phan G. . Structural and dynamical insights into the opening mechanism of *P. aeruginosa* OprM channel. Structure 18, 507–517 (2010). 20399187 10.1016/j.str.2010.01.018

[b13] Akama H. . Crystal structure of the membrane fusion protein, MexA, of the multidrug transporter in *Pseudomonas aeruginosa*. J. Biol. Chem. 279, 25939–25942 (2004). 15117957 10.1074/jbc.C400164200

[b14] Higgins M. K., Bokma E., Koronakis E., Hughes C. & Koronakis V. Structure of the periplasmic component of a bacterial drug efflux pump. Proc. Natl Acad. Sci. USA 101, 9994–9999 (2004). 15226509 10.1073/pnas.0400375101 PMC454203

[b15] Sennhauser G., Bukowska M. A., Briand C. & Grütter M. G. Crystal structure of the multidrug exporter MexB from Pseudomonas aeruginosa. J. Mol. Biol. 389, 134–145 (2009). 19361527 10.1016/j.jmb.2009.04.001

[b16] Symmons M. F., Bokma E., Koronakis E., Hughes C. & Koronakis V. The assembled structure of a complete tripartite bacterial multidrug efflux pump. Proc. Natl Acad. Sci. USA 106, 7173–7178 (2009). 19342493 10.1073/pnas.0900693106 PMC2678420

[b17] Eicher T. . Coupling of remote alternating-access transport mechanisms for protons and substrates in the multidrug efflux pump AcrB. eLife 3, e03145 (2014). 10.7554/eLife.03145 PMC4359379 25248080

[b18] Fernandez-Recio J. . A model of a transmembrane drug-efflux pump from Gram-negative bacteria. FEBS Lett. 578, 5–9 (2004). 15581607 10.1016/j.febslet.2004.10.097

[b19] Husain F., Humbard M. & Misra R. Interaction between the TolC and AcrA proteins of a multidrug efflux system of *Escherichia coli*. J. Bacteriol. 186, 8533–8536 (2004). 15576805 10.1128/JB.186.24.8533-8536.2004 PMC532411

[b20] Pos K. M. Drug transport mechanism of the AcrB efflux pump. Biochim. Biophys. Acta 1794, 782–793 (2009). 19166984 10.1016/j.bbapap.2008.12.015

[b21] Tal N. & Schuldiner S. A coordinated network of transporters with overlapping specificities provides a robust survival strategy. Proc. Natl Acad. Sci. USA 106, 9051–9056 (2009). 19451626 10.1073/pnas.0902400106 PMC2690002

[b22] Ruggerone P., Murakami S., Pos K. M. & Vargiu A. V. RND efflux pumps: structural information translated into function and inhibition mechanisms. Curr. Top. Med. Chem. 13, 3079–3100 (2013). 24200360 10.2174/15680266113136660220

[b23] Ruggerone P., Vargiu A. V., Collu F., Fischer N. & Kandt C. Molecular dynamics computer simulations of multidrug RND efflux pumps. Comput. Struct. Biotechnol. J. 5, e201302008 (2013). 24688701 10.5936/csbj.201302008 PMC3962194

[b24] Schulz R., Vargiu A. V., Collu F., Kleinekathöfer U. & Ruggerone P. Functional rotation of the transporter AcrB: insights into drug extrusion from simulations. PLoS Comput. Biol. 6, e1000806 (2010). 20548943 10.1371/journal.pcbi.1000806 PMC2883587

[b25] Lomovskaya O. . Identification and characterization of inhibitors of multidrug resistance efflux pumps in *Pseudomonas aeruginosa*: novel agents for combination therapy. Antimicrob. Agents Chemother. 45, 105–116 (2001). 11120952 10.1128/AAC.45.1.105-116.2001 PMC90247

[b26] Nakashima R. . Structural basis for the inhibition of bacterial multidrug exporters. Nature 500, 102–106 (2013). 23812586 10.1038/nature12300

[b27] Opperman T. J. . Characterization of a novel pyranopyridine inhibitor of the AcrAB efflux pump of *Escherichia coli*. Antimicrob. Agents Chemother. 58, 722–733 (2014). 24247144 10.1128/AAC.01866-13 PMC3910843

[b28] Vargiu A. V., Ruggerone P., Opperman T. J., Nguyen S. T. & Nikaido H. Molecular mechanism of MBX2319 inhibition of *Escherichia coli* AcrB multidrug efflux pump and comparison with other inhibitors. Antimicrob. Agents Chemother. 58, 6224–6234 (2014). 25114133 10.1128/AAC.03283-14 PMC4187987

[b29] Tikhonova E. B., Dastidar V., Rybenkov V. V. & Zgurskaya H. I. Kinetic control of TolC recruitment by multidrug efflux complexes. Proc. Natl Acad. Sci. USA 106, 16416–16421 (2009). 19805313 10.1073/pnas.0906601106 PMC2752513

[b30] Tikhonova E. B., Yamada Y. & Zgurskaya H. I. Sequential mechanism of assembly of multidrug efflux pump AcrAB-TolC. Chem. Biol. 18, 454–463 (2011). 21513882 10.1016/j.chembiol.2011.02.011 PMC3082741

[b31] Touzé T. . Interactions underlying assembly of the *Escherichia coli* AcrAB-TolC multidrug efflux system. Mol. Microbiol. 53, 697–706 (2004). 15228545 10.1111/j.1365-2958.2004.04158.x

[b32] Su C.-C. . Crystal structure of the CusBA heavy-metal efflux complex of Escherichia coli. Nature 470, 558–562 (2011). 21350490 10.1038/nature09743 PMC3078058

[b33] Trépout S. . Structure of reconstituted bacterial membrane efflux pump by cryo-electron tomography. Biochim. Biophys. Acta 1798, 1953–1960 (2010). 20599691 10.1016/j.bbamem.2010.06.019

[b34] Tamura N., Murakami S., Oyama Y., Ishiguro M. & Yamaguchi A. Direct interaction of multidrug efflux transporter AcrB and outer membrane channel TolC detected via site-directed disulfide cross-linking. Biochemistry 44, 11115–11121 (2005). 16101295 10.1021/bi050452u

[b35] Weeks J. W., Celaya-Kolb T., Pecora S. & Misra R. AcrA suppressor alterations reverse the drug hypersensitivity phenotype of a TolC mutant by inducing TolC aperture opening. Mol. Microbiol. 75, 1468–1483 (2010). 20132445 10.1111/j.1365-2958.2010.07068.x PMC2875072

[b36] Du D. . Structure of the AcrAB-TolC multidrug efflux pump. Nature 509, 512–515 (2014). 24747401 10.1038/nature13205 PMC4361902

[b37] Hobbs E. C., Yin X., Paul B. J., Astarita J. L. & Storz G. Conserved small protein associates with the multidrug efflux pump AcrB and differentially affects antibiotic resistance. Proc. Natl Acad. Sci. USA 109, 16696–16701 (2012). 23010927 10.1073/pnas.1210093109 PMC3478662

[b38] Kim J.-S. . Structure of the tripartite multidrug efflux pump AcrAB-TolC suggests an alternative assembly mode. Mol. Cells 38, 180–186 (2015). 26013259 10.14348/molcells.2015.2277 PMC4332038

[b39] Denisov I. G., Grinkova Y. V., Lazarides A. A. & Sligar S. G. Directed self-assembly of monodisperse phospholipid bilayer Nanodiscs with controlled size. J. Am. Chem. Soc. 126, 3477–3487 (2004). 15025475 10.1021/ja0393574

[b40] Ritchie T. K. . Chapter 11 - Reconstitution of membrane proteins in phospholipid bilayer nanodiscs. Methods Enzymol. 464, 211–231 (2009). 19903557 10.1016/S0076-6879(09)64011-8 PMC4196316

[b41] Lambert O. . Trimeric structure of OprN and OprM efflux proteins from *Pseudomonas aeruginosa*, by 2D electron crystallography. J. Struct. Biol. 150, 50–57 (2005). 15797729 10.1016/j.jsb.2005.01.001

[b42] Janganan T. K. . Evidence for the assembly of a bacterial tripartite multidrug pump with a stoichiometry of 3:6:3. J. Biol. Chem. 286, 26900–26912 (2011). 21610073 10.1074/jbc.M111.246595 PMC3143649

[b43] Krishnamoorthy G., Tikhonova E. B. & Zgurskaya H. I. Fitting periplasmic membrane fusion proteins to inner membrane transporters: mutations that enable Escherichia coli AcrA to function with *Pseudomonas aeruginosa* MexB. J. Bacteriol. 190, 691–698 (2008). 18024521 10.1128/JB.01276-07 PMC2223704

[b44] Vaccaro L., Koronakis V. & Sansom M. S. P. Flexibility in a drug transport accessory protein: molecular dynamics simulations of MexA. Biophys. J. 91, 558–564 (2006). 16648168 10.1529/biophysj.105.080010 PMC1483075

[b45] Bokma E., Koronakis E., Lobedanz S., Hughes C. & Koronakis V. Directed evolution of a bacterial efflux pump: adaptation of the *E. coli* TolC exit duct to the *Pseudomonas* MexAB translocase. FEBS Lett. 580, 5339–5343 (2006). 16979625 10.1016/j.febslet.2006.09.005

[b46] Tikhonova E. B., Wang Q. & Zgurskaya H. I. Chimeric analysis of the multicomponent multidrug efflux transporters from gram-negative bacteria. J. Bacteriol. 184, 6499–6507 (2002). 12426337 10.1128/JB.184.23.6499-6507.2002 PMC135444

[b47] Janganan T. K., Bavro V. N., Zhang L., Borges-Walmsley M. I. & Walmsley A. R. Tripartite efflux pumps: energy is required for dissociation, but not assembly or opening of the outer membrane channel of the pump. Mol. Microbiol. 88, 590–602 (2013). 23565750 10.1111/mmi.12211 PMC3664412

[b48] Lomovskaya O., Zgurskaya H. I., Totrov M. & Watkins W. J. Waltzing transporters and ‘the dance macabre' between humans and bacteria. Nat. Rev. Drug Discov. 6, 56–65 (2007). 17159924 10.1038/nrd2200

[b49] Nikaido H. & Pagès J.-M. Broad-specificity efflux pumps and their role in multidrug resistance of Gram-negative bacteria. FEMS Microbiol. Rev. 36, 340–363 (2012). 21707670 10.1111/j.1574-6976.2011.00290.x PMC3546547

[b50] Yum S. . Crystal structure of the periplasmic component of a tripartite macrolide-specific efflux pump. J. Mol. Biol. 387, 1286–1297 (2009). 19254725 10.1016/j.jmb.2009.02.048

[b51] Morita Y., Tomida J. & Kawamura Y. MexXY multidrug efflux system of Pseudomonas aeruginosa. Front. Microbiol. 3, 408 (2012). 23233851 10.3389/fmicb.2012.00408 PMC3516279

[b52] Mima T., Sekiya H., Mizushima T., Kuroda T. & Tsuchiya T. Gene cloning and properties of the RND-type multidrug efflux pumps MexPQ-OpmE and MexMN-OprM from *Pseudomonas aeruginosa*. Microbiol. Immunol. 49, 999–1002 (2005). 16301811 10.1111/j.1348-0421.2005.tb03696.x

[b53] Pos K. M. & Diederichs K. Purification, crystallization and preliminary diffraction studies of AcrB, an inner-membrane multi-drug efflux protein. Acta Crystallogr. D Biol. Crystallogr 58, 1865–1867 (2002). 12351840 10.1107/s0907444902013963

[b54] Mokhonov V. . Multidrug transporter MexB of Pseudomonas aeruginosa: overexpression, purification, and initial structural characterization. Protein Expr. Purif. 40, 91–100 (2005). 15721776 10.1016/j.pep.2004.10.002

[b55] Ferrandez Y. . Amphipol-mediated screening of molecular orthoses specific for membrane protein targets. J. Membr. Biol. 247, 925–940 (2014). 25086771 10.1007/s00232-014-9707-3

