# Supplementary material for: Tripartite assembly of RND multidrug efflux pumps
Source: Nat Commun. 2016 Feb 12;7:10731. doi: 10.1038/ncomms10731 (PMC4754349; doi:10.1038/ncomms10731)
Supplement: Supplementary Information — Supplementary Figures 1-7 [file ncomms10731-s1.pdf]

Supplementary Figure 1

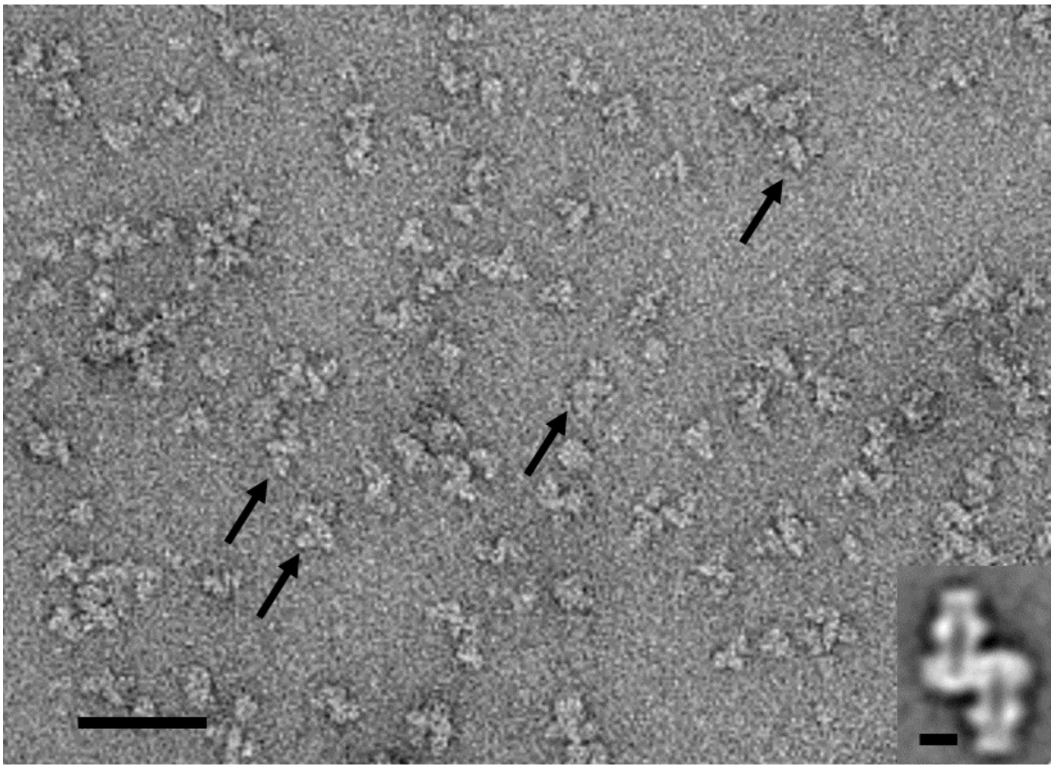

TEM observation of OprM reconstituted into nanodiscs with MSP1E3D1.

Field of view showing side views of one or two OprM molecules per ND (black arrows). The average image (inset) reveals two OprM molecules inserted in opposite orientations into the ND (215 images). The diameter of ND can accommodate two OprM molecules because of the larger size of MSP1E3D1 compared with MSP1D1 (inset). Scale bars 50 nm and 5 nm.

Supplementary Figure 2

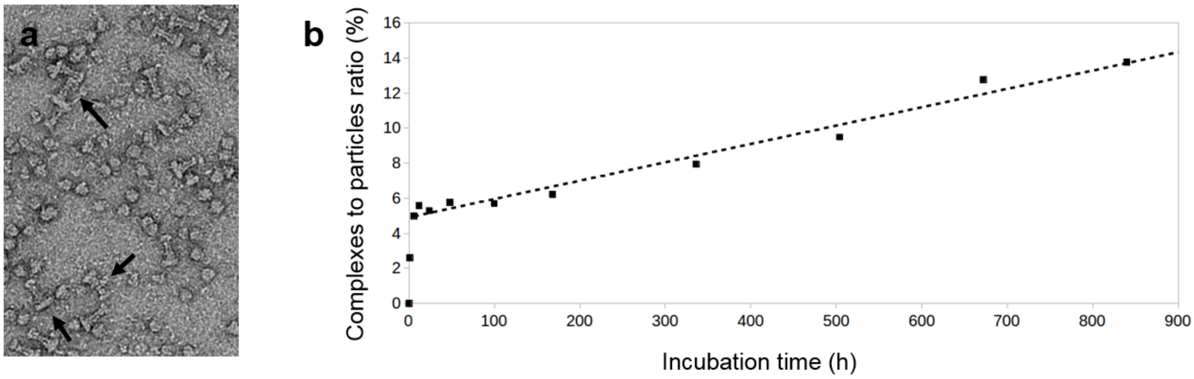

Tripartite complex formation over time

(a) After component mixing, the formation of tripartite complexes has been monitored over time by estimating the percentage of tripartite complexes with respect to the total particle population visible on EM grids. (b) This analysis shows that approx. 2.5% of tripartite complexes were formed within 1 hour after mixing of the components. After 6 hours, 5% of all observed particles on the grid were tripartite complexes. After this initial fast increase of tripartite complex formation, a slower and gradual increase (from approx. 5 % to 14 %) of complex particles was observed for samples taken at times indicated (up to 6 weeks). Dashed line: linear regression of the sample points.

Supplementary Figure 3

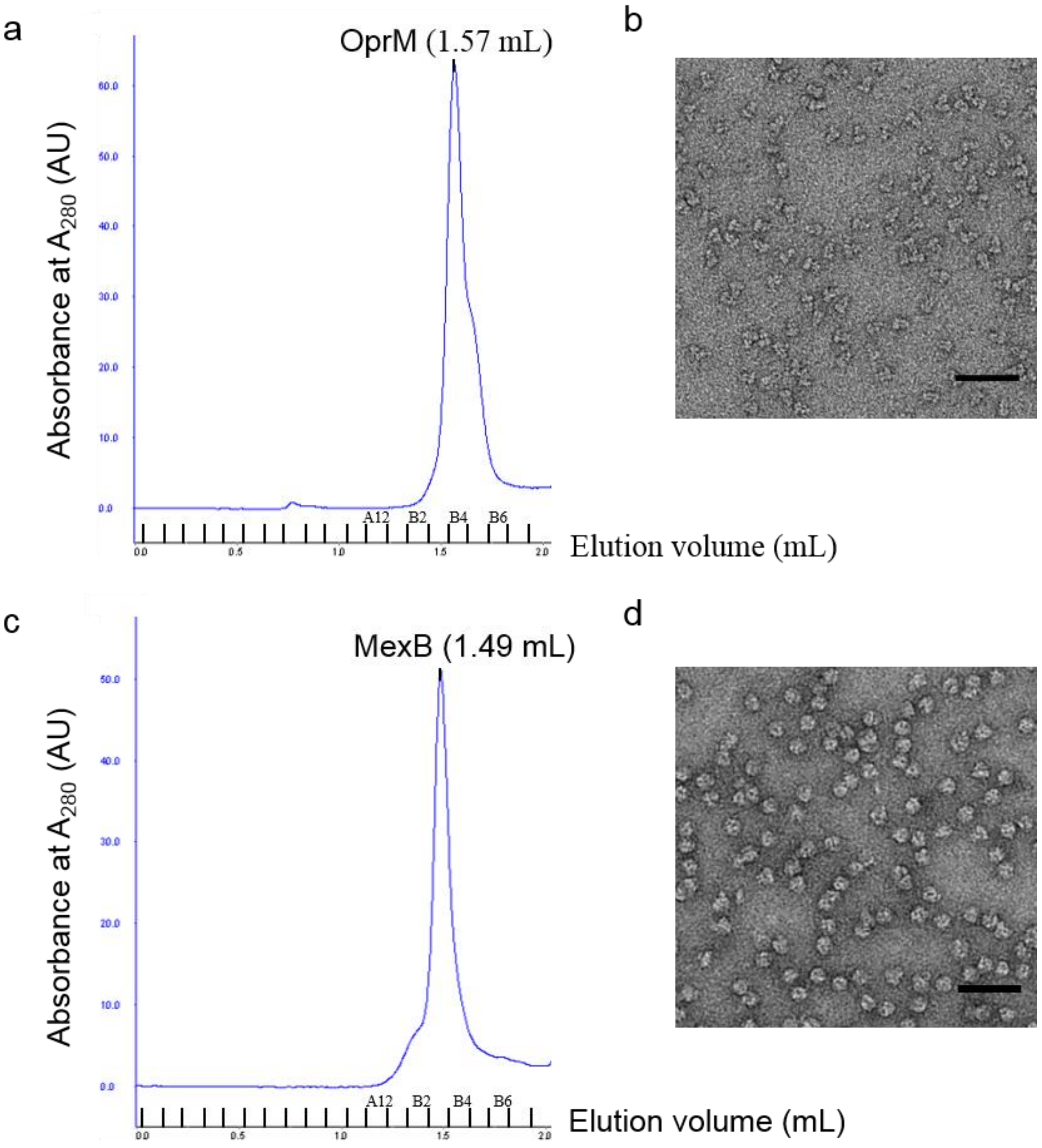

Purification of the OprM-ND and MexB-ND

(a-c) Analytical size-exclusion chromatography (SEC) analysis on a Superose 6 column of OprM-ND and MexB respectively. **(b, d)** TEM observations of B4 fraction and B3 fraction showing OprM-ND and MexB-ND molecules respectively. Scale bar 50 nm.

Supplementary Figure 4

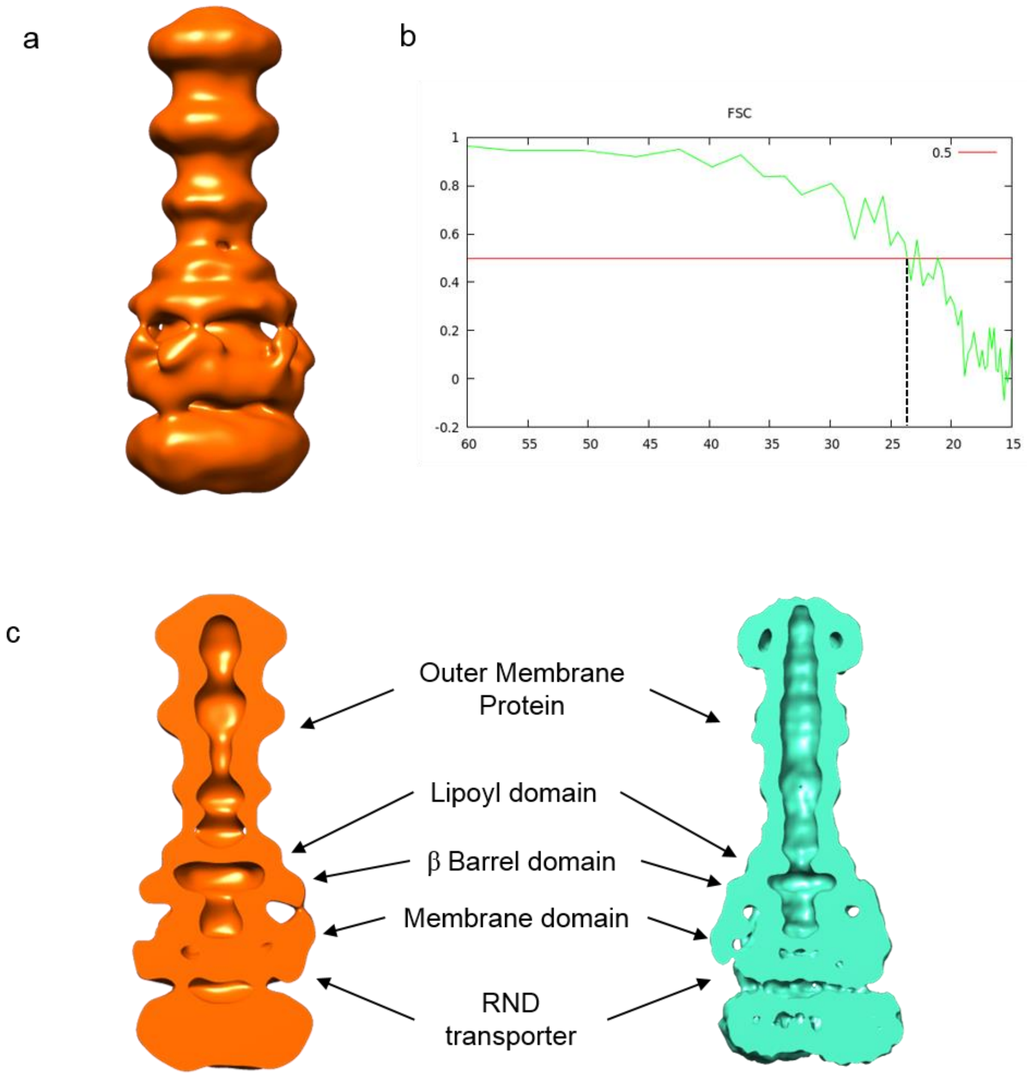

3D Reconstruction of the MexAB-OprM tripartite assembly.

(a) 3D reconstruction from 3065 selected side views and 936 top views filtered up to 25 Å. Using Spider software, a first initial volume was calculated from randomly oriented side views and was used as a reference in an iterative refinement process. A C3 symmetry was applied in this second step. **(b)** The resolution of the 3D reconstruction was estimated at 25 Å using Fourier shell correlation 0.5 cutoff criterion (the resolution where the *FSC* value falls to 0.5). The FSC curve was calculated between reconstructions from two halves of the data. **(c)** Cut away views of the EM map (orange) and the cryoEM reconstruction, EMD-5915 (cyan). Our 3D EM isosurface is in a good agreement with the cryoEM isosurface of the chimeric assembly (Du et al., 2014).

Supplementary Figure 5

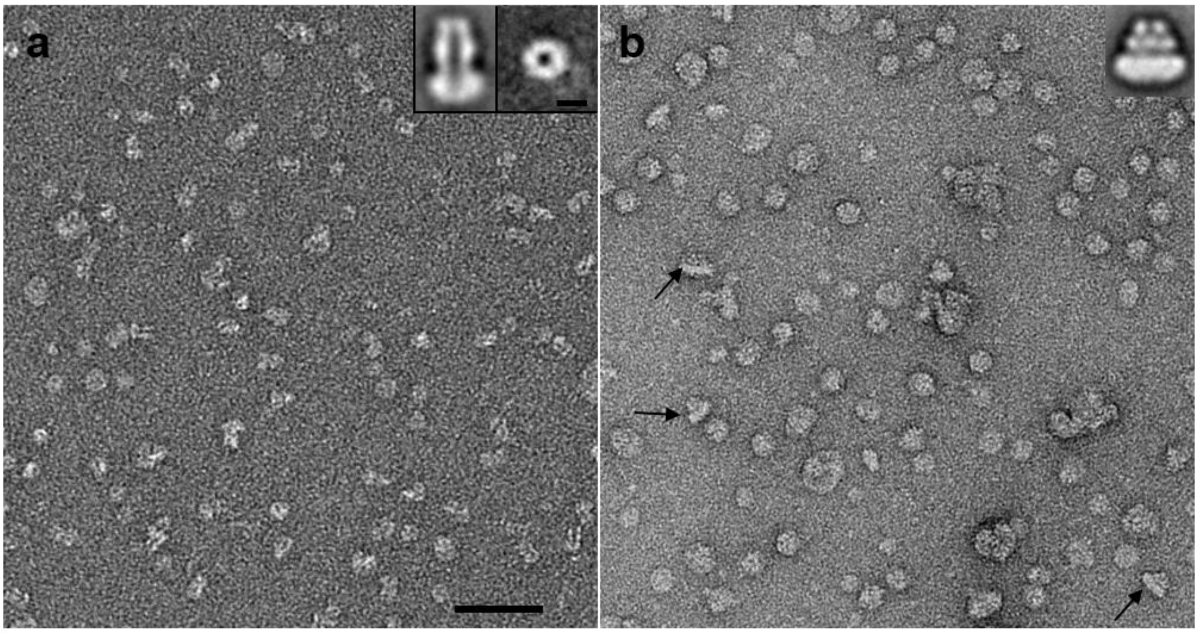

TEM observations of TolC and AcrB reconstituted into nanodiscs.

(**a**) Field of view of TolC-ND showing side and top views of isolated molecules. Inset: The average images of side (444 images) and top views (310 images) reveal characteristic features: TolC β-barrel embedded in the ND (bottom part of the image) and the TolC periplasmic domain protruding from the ND (top part of the image). (**b**) Field of view of AcrB-ND showing isolated molecules (black arrows). On the average image (inset), a side view of AcrB exhibiting the ND-embedded transmembrane domain (bottom part of the image) and the periplasmic part (top part of the image) resembling the features of the MexB periplasmic porter and funnel domains (Fig. 2). Scale bars 50 nm and 5 nm for the inset.

Supplementary Figure 6

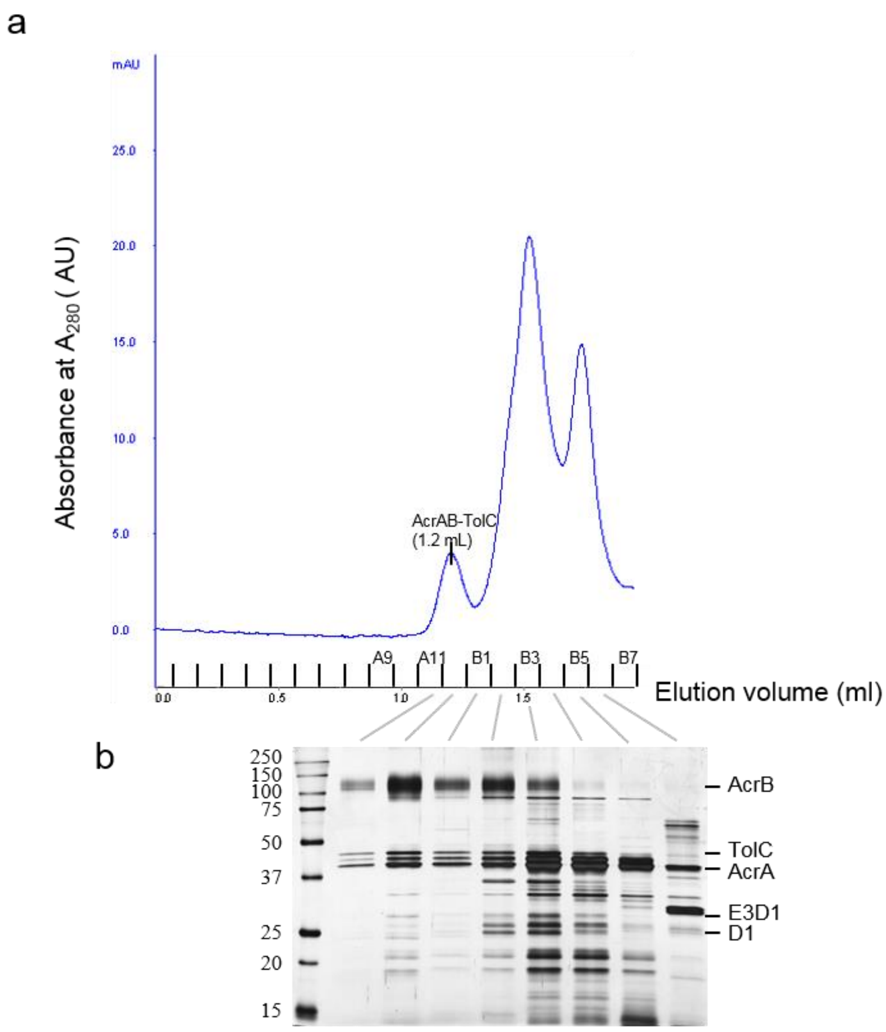

Purification of the tripartite AcrAB-TolC assembly in nanodiscs

(a) Analytical size-exclusion chromatography (SEC) analysis of mixed AcrA, AcrB, and TolC components. **(b)** SDS-PAGE analysis of the indicated SEC peak fractions. The molecular mass of each marker protein (in kilodalton) is indicated on the left.

Supplementary Figure 7

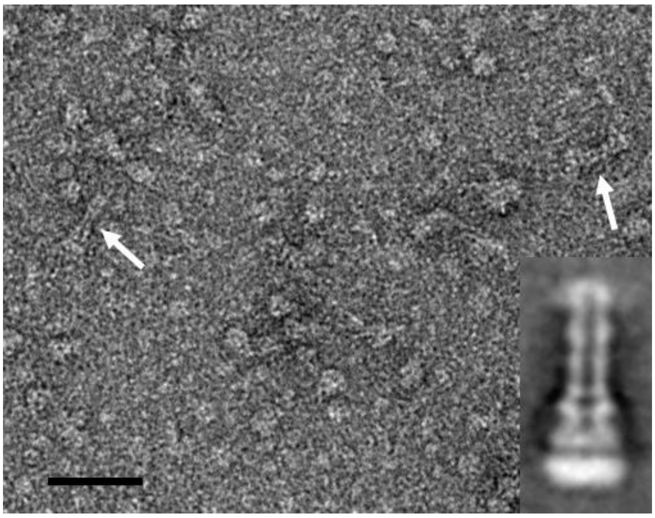

TEM observation of tripartite AcrA-MexB-TolC assembly in nanodiscs

Field of view revealing elongated complexes (white arrows) when TolC-ND and MexB-ND were mixed in the presence of AcrA. Scale bar 50 nm. **Inset:** Average image of a 33 nm long tripartite AcrA-MexB-TolC complex delineated by two nanodiscs. The isocontours of this complex are shown in Fig. 5d.
